# Differential Effects of Tissue-Specific Deletion of BOSS on Feeding Behaviors and Energy Metabolism

**DOI:** 10.1371/journal.pone.0133083

**Published:** 2015-07-20

**Authors:** Ayako Kohyama-Koganeya, Mizuki Kurosawa, Yoshio Hirabayashi

**Affiliations:** Molecular Membrane Neuroscience, Brain Science Institute, RIKEN, Wako-shi, Saitama, Japan; Kobe University, JAPAN

## Abstract

Food intake and energy metabolism are tightly controlled to maintain stable energy homeostasis and healthy states. Thus, animals detect their stored energy levels, and based on this, they determine appropriate food intake and meal size. *Drosophila melanogaster* putative G protein-coupled receptor, Bride of sevenless (BOSS) is a highly evolutionarily conserved protein that responds to extracellular glucose levels in order to regulate energy homeostasis. To address how BOSS regulates energy homeostasis, we characterized a *boss* mutant by assessing its food intake and stored energy levels. *Boss* mutants exhibited increased food intake but decreased stored triacylglyceride levels. Using boss-GAL4 drivers, we found that *boss* is expressed in select tissues that are involved in nutrient sensing and food intake, in a subset of neurons in brain and chemosensory organs, in fat body, and in endocrine cells in gut (enteroendocrine cells). Flies with tissue-specific *boss* knockdowns in these tissues had abnormal stored energy levels and abnormal food intake. These results suggest that BOSS in either neurons or peripheral nutrient-sensing tissues affects energy homeostasis in ways that relate to the sensing of nutrients and regulation of food intake.

## Introduction

To maintain energy homeostasis, energy sensing tissues, such as brain, gut, and adipose tissue, translate changes in internal metabolic state into alterations in feeding behavior [1]. Understanding how these tissues communicate with each other is fundamental for understanding state-dependent modifications of behavior and also for elucidating the relevance of this communication to human obesity and associated diabetes and cardiovascular disease. *Drosophila melanogaster*, a widely used genetic model animal, provides a useful model system to study feeding behavior, because many molecules involved in the regulation of energy homeostasis are highly conserved between flies and mammals [2].

*Drosophila* BOSS is an orphan G protein-coupled receptor (GPCR) membrane protein that was first identified as a ligand for sevenless tyrosine kinase (SEVENLESS), which is involved in eye differentiation in *Drosophila* [3, 4]. Beside this function, we found that BOSS has an additional critical function: to respond to extracellular glucose levels and regulate energy homeostasis [5]. This function seems to be conserved during evolution, because knockout of the mice homologue GPRC5B protected the mice from obesity resulting from a high-fat diet [6, 7].

We previously reported that energy homeostasis is disrupted in a *boss* mutant, suggesting that BOSS may play a role in regulating food intake and energy metabolism [5]. Thus, we examined food intake and further metabolic phenotypes of *boss* mutant flies. We found that stored lipid levels are reduced, but food intake is increased in *boss* mutant flies. To elucidate how BOSS regulates food intake and energy metabolism, we first analyzed tissue-specific BOSS expression in *Drosophila*. BOSS is expressed in neurons of chemosensory organs and in the brain, in enteroendocrine cells in the gut, and in the fat body. Then, we generated tissue-specific *boss* knockdowns and showed that BOSS either in neurons or in the peripheral tissues (the fat body and enteroendocrine cells) regulates energy stores and food intake. Our findings suggest that BOSS is an important regulator of energy homeostasis in nutrient-sensing tissues.

## Materials and Methods

### Fly stocks and genetics

*Boss-GAL4* lines: A 2.1kbp DNA fragment upstream of the putative transcriptional start site of *boss* [8] was subcloned into a pUAST vector (Drosophila Genomics Resource Center) and microinjected into w embryos (BestGene). Wild-type w^1118^ wild-type flies were used as controls. The following lines were also used: elav-Gal4, 10xUAS-mCD8GFP, UAS-mCherry^NLS^ (Bloomington Drosophila Stock Center, Indiana University, Bloomington, IN, USA); FB-Gal4 (gift from Dr. R. Kuhnlein, Max Planck Institute for Biophysical Chemistry, Gottingen, Germany); prospero-Gal4 (gift from Dr. G. Suh, Skirball Institute of Biomolecular Medicine, NYU, New York, NY, USA); UAS-boss RNAi (#4365 and #4366, VDRC, Vienna, Austria). To visualize BOSS expression patterns, 10XUAS-mCDGFP/+;Boss-Gal4/+ and 10X UAS-mCD8GFP/Boss-Gal4, UAS-mCherry^NLS^ were produced by crossing 10X UAS-mCD8GFP to Boss-Gal4 or Boss-Gal4, UAS-mCherry^NLS^. To generate tissue specific *boss* knockdown flies, adult flies carrying different Gal4 driver (elav-Gal4 for neuronal, prospero-Gal4 for enteroendocrine cells and FB-Gal4 for fat body knockdown) were crossed to boss-RNAi lines [boss-RNAi#1(#4365, on the second chromosome) or boss-RNAi#2 (#4366, on the third chromosome)].

### Husbandry

*Drosophila melanogaster* were grown using standard food comprising 10% glucose, 4% yeast, 4% corn meal, and 1% agar. They were maintained at 25°C, under a 12-hour light and 12-hour dark regime. Flies were collected at the middle of light regime and used for experiments.

### Food intake Assays

For the blue-dye food assay, male flies aged 3–5 days were switched from standard food to blue-colored food (10% sucrose, 1% agar, 2% blue no.1 dye) for 1 h. After the feeding, 10 flies were immediately frozen in one tube using liquid nitrogen. Then, flies were homogenized in PBS buffer and centrifuged (13000 rpm) for 15 min. The supernatants were transferred to a new tube, and absorbance was measured at 625 nm.

We used a capillary feeder (CAFE) to measure ingestion by flies. For the CAFE assay, 10 male flies (aged 3–5 days) that were fed on standard food were transferred to an experimental vial containing a glass micropipette and allowed to feed on 10% sucrose solution. The amount consumed, minus evaporation (determined from a vial without flies), was measured. For the post-starvation response of the feeding assay, male flies starved for 18 h or 24 h in vials containing 1% agar were transferred to vials containing blue-colored food or CAFE assay vials.

### Metabolic assay for triacylglyceride (TAG) and glycogen

Male flies aged 3–5 days were homogenized in PBS. Supernatant was collected after heat inactivation at 70°C for 5 min and centrifugation at 13000 rpm for 5 min. TAG levels were measured using a TAG assay kit (Sigma, TR0100). Glycogen levels were measured using a glycogen assay kit (Sigma, MAK016). Data were normalized to the total protein measured by a Bradford assay.

### Hemolymph sugar measurements

Hemolymph from five groups each comprising 20 female flies aged 3–5 days was used for each condition. Hemolymph was diluted (1:10) in homogenization buffer (137 mM NaCl, 2.7 mM KCl, 5 mM Tris [pH 6.6]) and heated for 5 min at 70°C. Glucose and trehalose levels were measured using a glucose assay kit (Sigma, GAGO-20). To measure trehalose levels in the samples, porcine trehalase (Sigma, T8778) was used to convert trehalose into glucose and quantified with glucose assay kit. The background level of glucose present in the sample was subtracted.

### Immunofluorescence

Larvae and flies were dissected in PBS, fixed in PBS containing 4% formaldehyde for 25 min at room temperature, and washed in PBS containing 0.2% Triton X-100 (PBT). Tissues were blocked for 15 min in Blocking One Histo (Nacalai Tesque Inc., Nakagyo-ku, Kyoto, Japan). Primary antibodies were incubated overnight at 4°C, and Alexa Fluor-conjugated secondary antibodies (Life Technologies) were incubated for 2 h at room temperature. Tissues were mounted in Vectashield mounting medium (Vector Laboratories, Inc.). Confocal images were collected using Olympus/FV1000 confocal system.

#### Antibodies

We used chick anti-GFP (1:500, Abcam); rat anti-RFP (1:500, [5F8] Chromotek); mouse anti-nc82 (1:20, DSHB); and mouse anti-prospero (1:50, DSHB).

### Quantitative real-time PCR (qRT-PCR)

RNA was collected from male flies aged 3–5 days. Samples (10 flies per sample) were immediately frozen in liquid nitrogen. Total RNA was extracted using Trizol Reagent (Invitrogen). Total RNA samples (1 μg per reaction) were reverse transcribed using oligo-dT and random primers and Superscript RT-III (Invitrogen). The generated cDNA was used for real-time RT-PCR (qPCR Mastermix Plus for SYBERGreen, Applied Biosystems). Three separate samples were collected from each condition, and triplicate measurements were conducted. Primers (S1 Table) were designed using the Universal Probe Library Assay Design Center (Roche Applied Science).

### Western blotting

Ten male flies aged 3–5 days were used for each condition. The bodies were dissected out in Schneider’s Drosophila medium and homogenized in sample buffer (Nacali Tesque Inc.). For insulin treatment, the bodies were incubated with or without 5 μM recombinant human insulin (Sigma, I2643) for 1 h at 25°C before being homogenized in sample buffer. Rabbit anti-pAKT (1:1000, Cell signaling); rabbit anti-AKT (1:1000, Cell signaling); and mouse anti-tubulin (1:10,000, Sigma) were used for primary antibodies, and HRP-conjugated anti-rabbit or anti-mouse IgG antibodies (Cell Signaling Technology) were used for secondary antibodies. Blots were visualized using the Chemi-Lumi One Super reagent (Nacali Tesque Inc.).

### Statistics

For all experiments, error bars represent standard error of the mean (SEM), and p values refer to results of a Student’s test performed in Microsoft Excel (version 14.4.4) (*p<0.05).

## Results

### *Boss* mutant exhibits abnormal energy homeostasis

**Fig 1:**
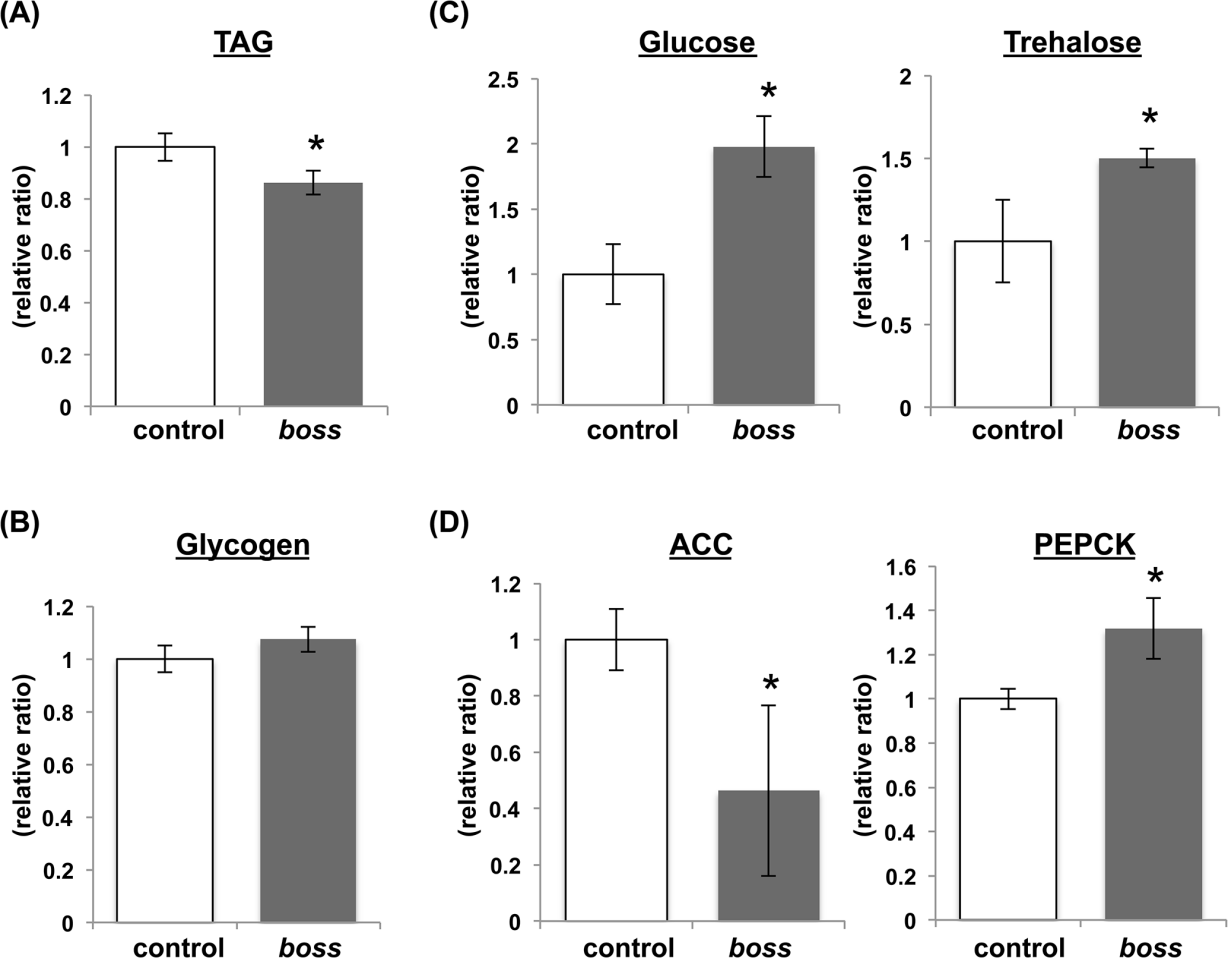
BOSS controls energy metabolism. (A and B) Relative lipid (A) and glycogen (B) content in WT and *boss* mutant adult flies (n = 3, 10 flies per replicate). WT, wild type; TAG, triacylglyceride. (C) Circulating sugar levels in the hemolymph of *boss* mutant adult flies. ACC, acetyl-CoA carboxylase (n = 3, 20 flies per replicate). (D) Amounts of *ACC* and *PEPCK* mRNA in *boss* mutant flies relative to control flies (n = 3, 10 flies per replicate). PEPCK, phosphoenolpyruvate carboxykinase. Data are represented as mean ±SEM (*P<0.05).

A previous study found that *boss* mutant larvae exhibit abnormal sugar and lipid metabolism (i.e., elevated circulating sugar and lipid levels and impaired lipid mobilization to oenocytes), and are sensitive to nutrient deprivation stress [5]. Thus, we first measured energy store (TAG and glycogen) levels in adult *boss* mutant flies. In normal *Drosophila*, TAG and glycogen constitute the major forms of energy storage for carbohydrates and lipids, respectively. Adult *boss* mutant flies had reduced TAG levels (Fig 1A), but no difference in glycogen levels (Fig 1B). We also measured hemolymph sugar levels in adult flies. The *boss* mutant had high trehalose and glucose sugar levels (Fig 1C), levels that are comparable to larval hemolymph sugar levels, which we previously measured [5].

Acetyl-CoA carboxylase [9] is the rate-limiting enzyme for fatty acid synthesis [9]. We found that ACC expression was reduced in *boss* mutant flies (Fig 1D). Furthermore, we found that expression of phosphoenolpyruvate carboxykinase (PEPCK), which catalyzes the rate-limiting step of gluconeogenesis and is used as a marker for starvation, was increased in the *boss* mutant flies (Fig 1D). Overall, these results demonstrate that energy metabolism is disrupted in the *boss* mutant.

### Insulin signaling pathway is downregulated in the *boss* mutant

**Fig 2:**
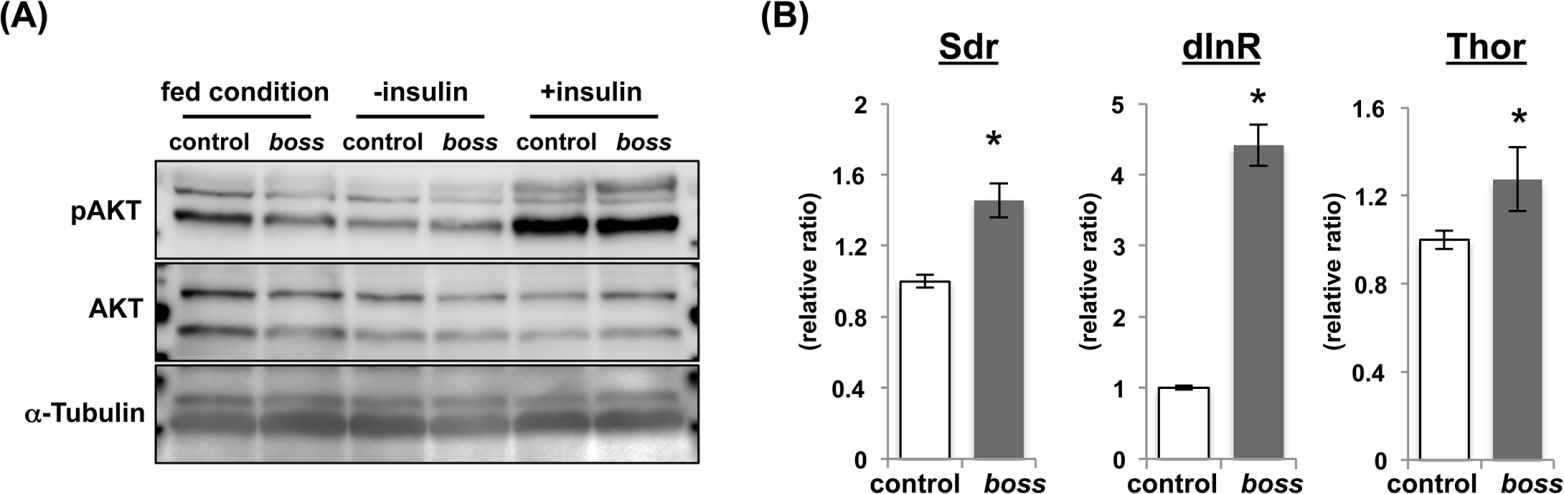
BOSS affects insulin signal activity. (A) Immunoblot detecting AKT phosphorylation. Without insulin stimulation, pAKT levels are decreased in *boss* mutant flies. AKT phosphorylation levels in *boss* mutant flies after insulin stimulation. (B) Amounts of *Sdr*, *dInR* and Thor mRNA in *boss* mutant flies relative to control flies (n = 3, 10 flies per replicate). Data are represented as mean ±SEM (*P<0.05).

Insulin signaling regulates lipid and glucose metabolism in *Drosophila* similarly to that in vertebrates [10]. In line with the aberrant lipid and glucose metabolism in *boss* mutant flies, we examined the activity of insulin signaling in these flies. First, we measured phosphorylated AKT (pAKT) levels. Under normal feeding conditions, the *boss* mutant flies had reduced pAKT levels (Fig 2A). With insulin stimulation, activated pAKT is used as an indication of insulin resistance [11]. Thus, we examined the ability of exogenous insulin to stimulate the phosphorylation of AKT. The *boss* mutant flies responded to insulin, leading to an increase in pAKT similar to that in control flies (Fig 2A). This *ex vivo* experiment demonstrates that the insulin-signaling machinery of *boss* mutant flies is functional but that the insulin-signaling is not activated *in vivo*. We speculated that *Drosophila* insulin-like peptides (DILPs) activity might be reduced. In *Drosophila*, eight DILPs (DILP1-8) are identified, and DILP2, 3, 5 and 6 have been shown to play a physiological role in metabolic control [12]. We, therefore, measured these four dilps’ mRNA expressions by using qRT-PCR. The amounts of mRNA for *dilp2* and *dilp3* were reduced, but those for *dilp5* and *dilp6* were increased in the *boss* mutant flies (S1 Fig). Regulation of dilps expression is very complicated [10, 13, 14]. For example, starvation reduces expression of *dilp3* and *dilp5*, but not *dilp2*. Moreover, knockdown of *dilp2* expression is compensated for by increases in *dilp3* and *dilp5*. Thus, further studies will be required to delineate whether BOSS directly coordinates the regulation of DILPs secretion.

Recently, secreted decoy of InR (SDR) was identified as a negative regulator of insulin signaling [15]. SDR has similar structure to the extracellular domain of insulin receptor, and antagonizes insulin signaling via this domain. SDR is constantly secreted into the hemolymph and binds to DILPs. In *boss* mutant flies, *Sdr* mRNA expression was increased (Fig 2B), indicating that increased *Sdr* expression is one of the causes of insulin signaling suppression in these flies.

To confirm insulin-signaling activity in the *boss* mutant flies, we examined the activity of dFOXO. Insulin signaling inhibits FOXO activity [10]. We found that expression of *Drosophila Insulin receptor* (*dInR*) and *Thor/4E-BP*, which are FOXO target genes, were reduced in the *boss* mutant (Fig 2B). Although it remains to be solved how insulin signaling is downregulated in the *boss* mutant, these data suggest that the insulin signaling pathway is inactivated in *boss* mutant flies.

### *Boss* mutant flies exhibit increased food intake

**Fig 3:**
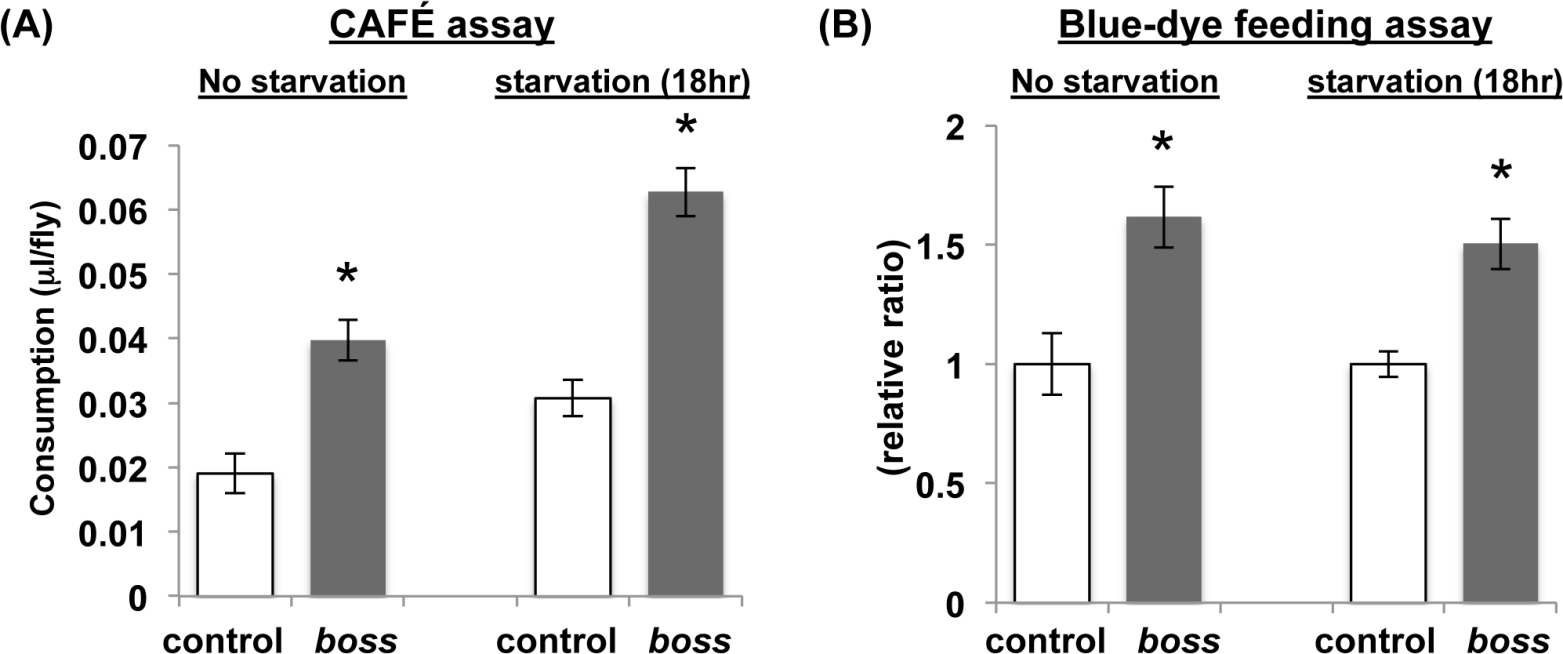
*Boss* mutant flies are hyperphagic. (A) Food consumption measured by the CAFE assay (n = 10, 10 flies per replicate). (B) Food consumption measured by Blue-dye ingestion assay. The ingestion of dye was quantified after feeding male flies for 1h in the light cycle, and then by measuring the absorbance of ingested dye (n = 10, 10 flies per replicate). Data are represented as mean ±SEM (*P<0.05).

Changes in food intake behavior can affect energy homeostasis in flies. Thus, we examined levels of food intake in the *boss* mutant by using the CAFE assay (Fig 3A) and blue-dye feeding assay (Fig 3B). *Boss* mutant flies consumed significantly more food than control flies under free-feeding and starving conditions. These results demonstrate that *boss* mutant flies display marked increases in food intake (Fig 3).

### BOSS is expressed in nutrient-sensing tissues

Food intake is a necessary behavior that provides energy to survive, and this depends on body energy balance. Tissues communicate with each other and strive to maintain this balance [2, 16]. To elucidate the mechanisms of BOSS signaling, it is important to closely examine BOSS-expressing tissues. Thus, we analyzed *boss* expression by various tissues using a *boss-GAL4* driver. We generated *boss-GAL4* transgenic flies that contain 2.1kb upstream of the predicted transcription start site of the *boss* gene [8], and characterized the expression pattern of this *Gal4* driver using UAS-mCD8GFP and UAS-mCherry^NLS^.

**Fig 4:**
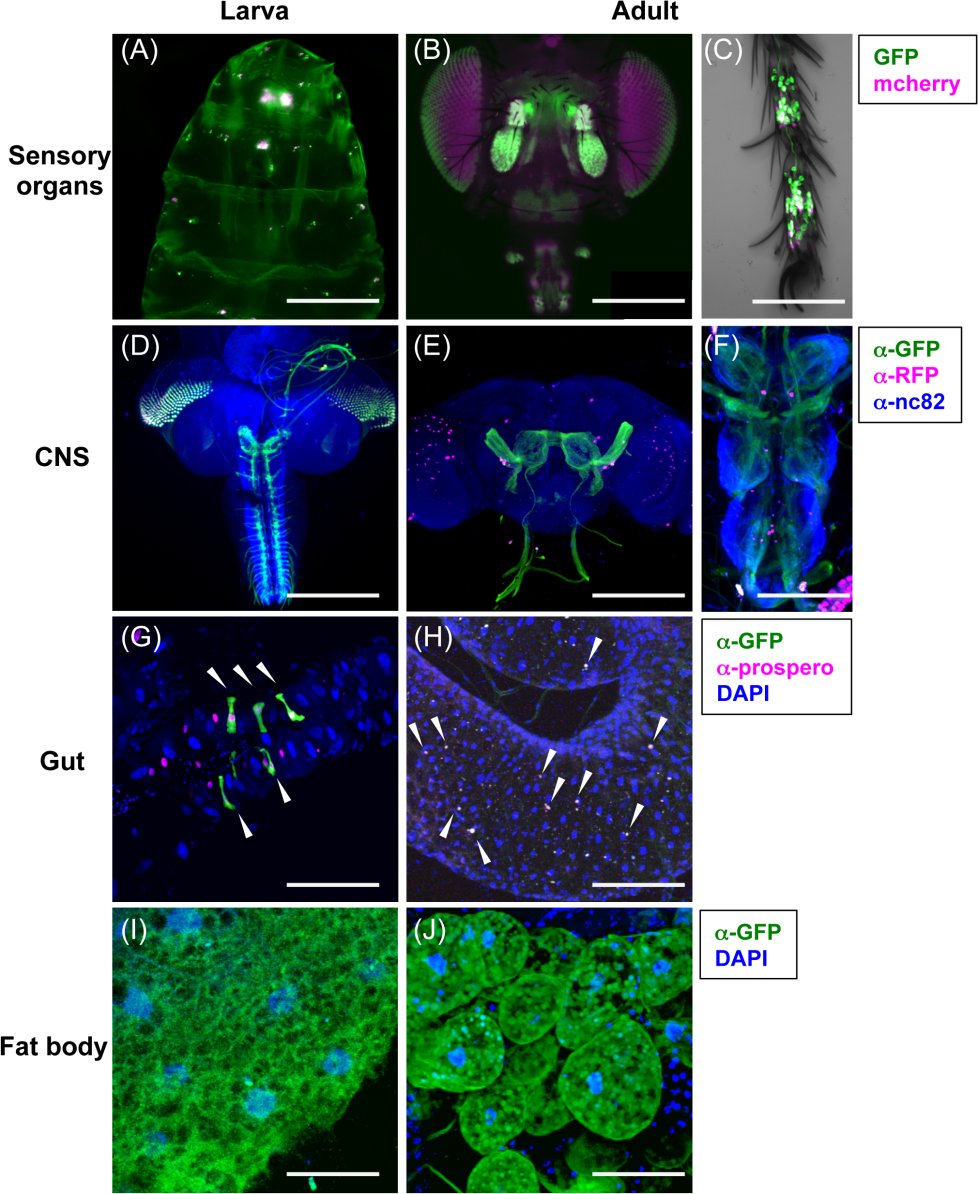
BOSS is expressed in nutrient-sensing tissues. Expression of *boss* in larval and adult flies was visualized by expression of 10xUAS-mCD8.GFP and UAS-mCherry^NLS^ using a *boss-GAL4* driver. (A-C) Expression in larval (A) and adult (B) sensory organs. (C) Adult foreleg. (D) Larval CNS. (E) Adult brain. (F) Adult thoracic ganglion. To identify the locations of *boss*-expressing cells, UAS-mCherry^NLS^ (magenta) was coexpressed with 10xUAS-mCD8.GFP [9] in the brain and sensory organs. The CNS was also labeled with antibody nc82 (blue), a marker that labels synapses. Larval (G) and adult (H) midgut. The gut was counterstained with DAPI (blue) and a marker for enteroendocrine cells, prospero (magenta). Arrowheads show the prospero-positive GFP-expressing cells. See also S2 Fig. Larval (I) and adult (J) fat body. The fat body was counterstained with DAPI (blue), a cell nucleus marker. Scale bars; 500 μm in A, 30 μm in B, 200 μm in C and E, 100 μm in G, H and J, 80 μm in D, 50 μm in H.

Live fluorescence imaging and immunohistochemistry showed that GFP and mCherry were expressed in tissues known to be involved in nutrient sensing and food intake (Fig 4). For example, GFP and mCherry expression were detected in chemosensory neurons of five specific chemosensory organs: the dorsal and ventral pharyngeal organ, the ventral pits of larvae, the antennae, the proboscis, and the legs of adults (Fig 4A–4C). Second, GFP and mCherry expression were detected in specific neurons of the central nervous system (CNS) (Fig 4D–4F). Third, GFP expression was detected in peripheral tissues, specifically, subsets of cells in the gut and the fat body (Fig 4G–4J). Most of these GFP-labeled cells in the midgut were co-labeled with the enteroendocrine cell marker prospero (93% in larva, and 98% in adult) (Fig 4G and 4H, S2 Fig), suggesting that *boss-GAL4* expressing cells in the gut are subsets of enteroendocrine cells.

CNS and enteroendocrine cells express and secrete hormones and neuropeptides, some of which are involved in energy metabolism [17] [18] [19]. Chemosensory organs detect nutrients and send signals to other tissues to coordinate food intake [20]. The fat body of flies, which is similar in function to mammalian liver and white adipose tissue, stores and releases energy in response to energy demands [21]. The BOSS expression profile we observed, therefore, indicates that BOSS coordinates inter-organ/inter-tissue communication to regulate food intake and energy metabolism. Therefore, we next examined the function of BOSS in these tissues.

### Tissue-specific *boss* knockdown flies exhibit contrasting food intake and metabolic phenotypes

**Fig 5:**
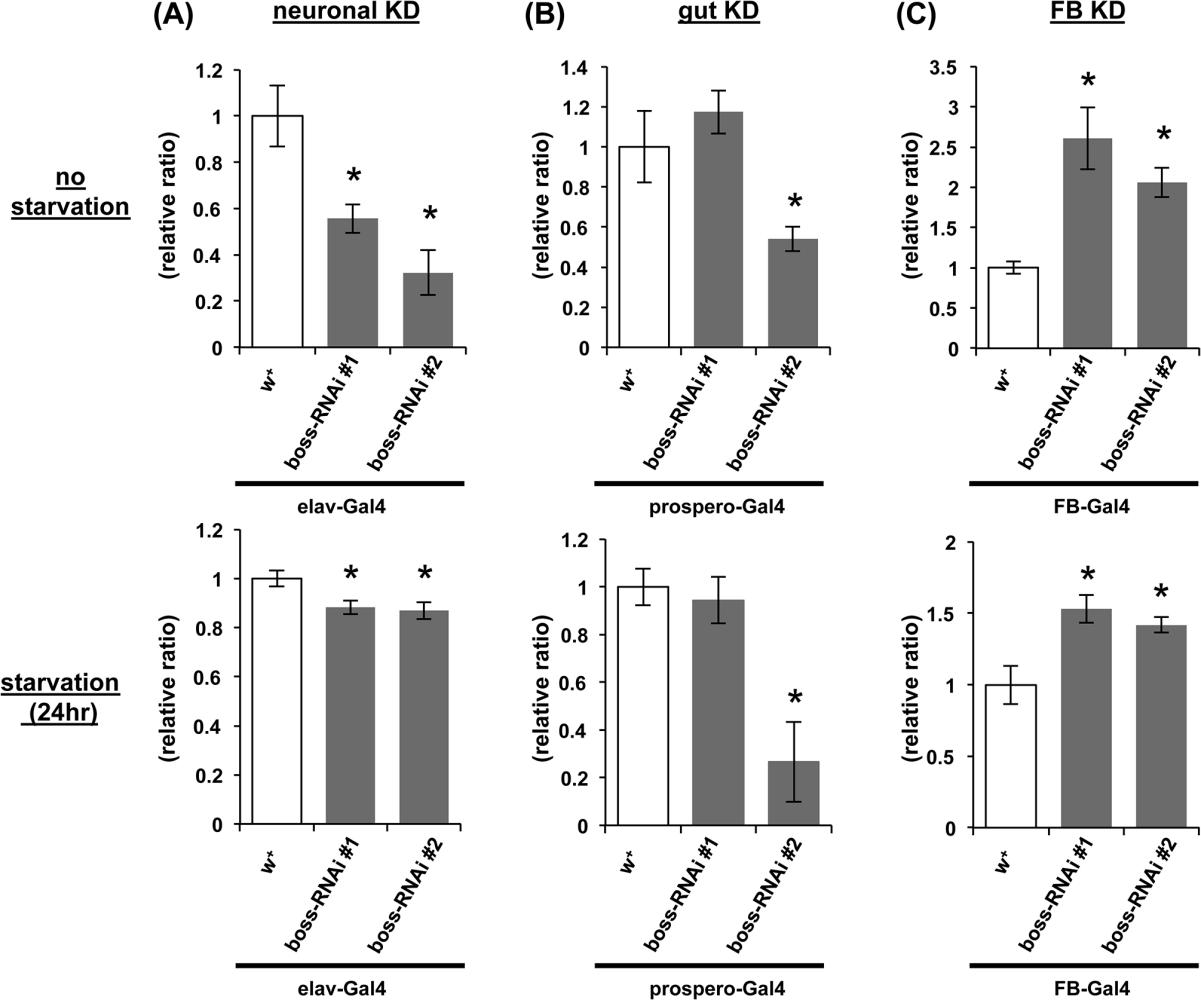
Effect of tissue-specific expression of boss RNAi on food intake. Flies expressing boss RNAi in neurons from the elav-GAL4 (A), enteroendocrine cells from proepero-GAL4 (B), and fat body from FB-GAL4 (C) were used for blue-dye ingestion assay. The ingestion of dye was quantified after feeding male flies for 1 h, and thereafter by measuring the absorbance of ingested dye. Two transgene insertions were used: boss-RNAi #1 and #2 (n = 10, 10 flies per replicate). Assays were performed under two conditions [no starvation (upper row) and 24h starvation (lower row)]. Data are represented as mean ±SEM (*P<0.05).

To determine the function of BOSS in the tissues, neurons, enteroendocrine cells and fat bodies, we generated tissue-specific *boss* knockdown flies (S3 Fig) and measured their food intake under free-feeding and starved states. Neuronal-specific knockdown flies ingested decreased amounts of food (Fig 5A). One line of enteroendocrine cells-specific knockdown flies(RNAi #2)also showed reduced food ingestion, but the other line(RNAi #1)showed no difference (Fig 5B). We speculate this discrepancy may occur by positional effects of RNAi lines, since they were generated using P-element random insertion method [22]. Thus, there is a possibility that food intake phenotype was diminished by positional effects in RNAi #1 line. We need further experiments to confirm that boss expressed in enteroendocrine cells affects food intake regulation. In contrast, fat body-specific knockdown flies significantly and selectively increased their food ingestion in both states (Fig 5C).

**Fig 6:**
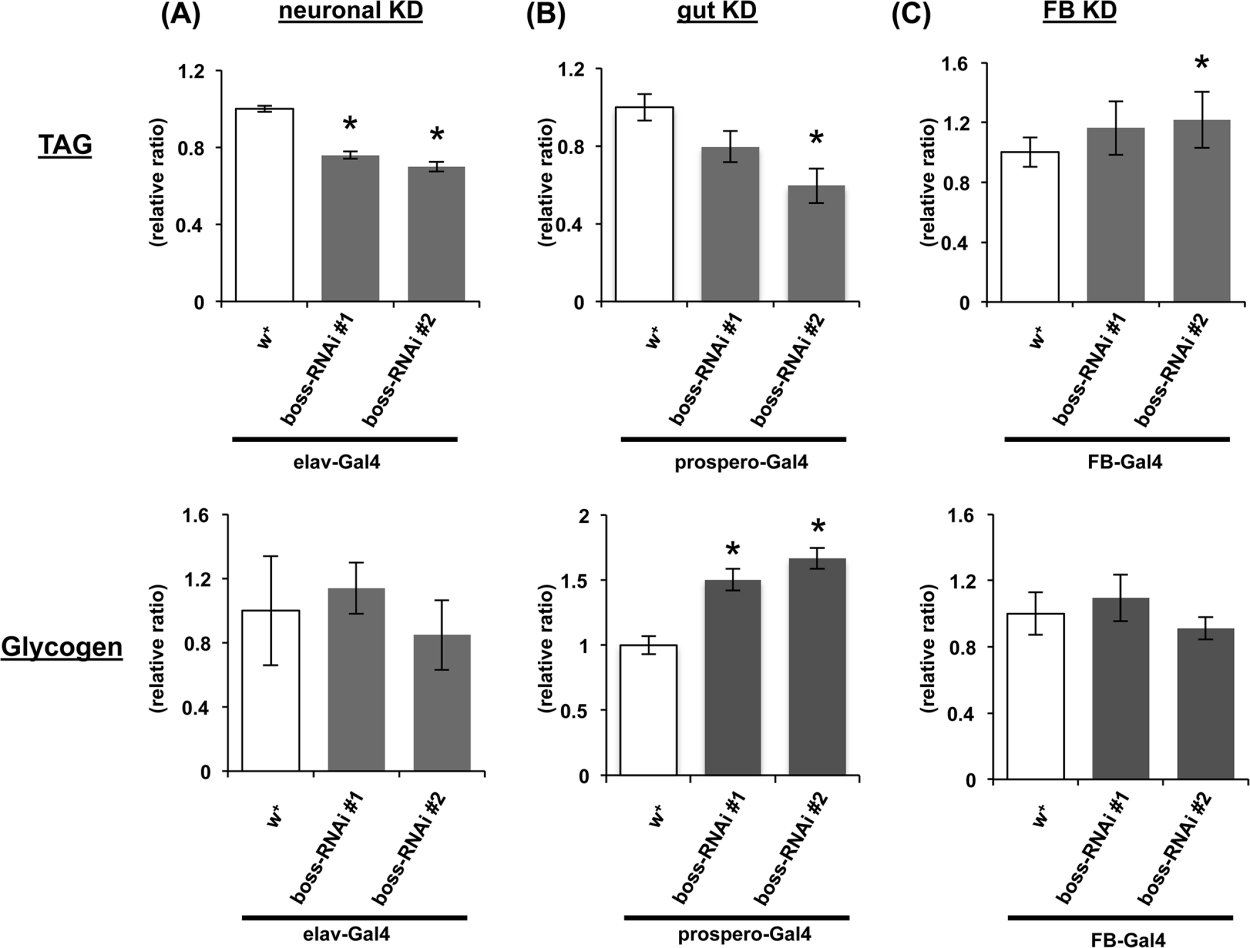
Effect of tissue-specific expression of boss RNAi on lipid and glycogen levels. TAG (upper row) and glycogen (lower row) levels were normalized to protein levels and expressed as ratios to the control. The flies’ genotypes are the same as those flies used in the blue-dye ingestion assay.

Next, we examined stored TAG and glycogen levels. Neuronal-specific knockdown flies had reduced stored TAG levels (Fig 6A and 6B). Enteroendocrine cells-specific knockdown flies had reduced stored TAG levels but increased stored glycogen levels (Fig 6C and 6D). On the other hand, fat body-specific knockdown flies had significantly increased stored TAG levels but not glycogen levels (Fig 6E and 6F). These results indicate that peripheral and neuronal BOSS is differentially involved in the regulation of energy stores.

## Discussion

The question of how animals control their feeding and energy metabolism and maintain energy homeostasis is one of the most important issues we currently need to understand. To approach this question, it is necessary to identify the molecule(s) that control and coordinate feeding and energy metabolism. Here, we present evidence that BOSS regulates food intake and energy metabolism. Without BOSS, food intake and energy storage levels in the body are altered, and energy homeostasis is disrupted. Furthermore, tissue-specific *boss* knockdown mutants flies exhibited the opposite food intake and metabolic phenotypes, indicating that BOSS coordinates the interaction between metabolic tissues for maintaining energy homeostasis (S4 Fig).

### BOSS contributes to energy homeostasis in tissues where it is expressed

We have shown that a *boss* null mutant and tissue-specific *boss* knockdown flies show aberrant energy metabolism (Figs 1 and 2) and food intake (Fig 3). One of the more important findings is that the phenotype of the fat-body-specific *boss* knockdown flies is opposite to that of the neuronal-specific or enteroendocrine-cell-specific *boss* knockdown flies. The fat-body-specific *boss* knockdown flies increased stored lipids and food intake. In contrast, the neuronal-specific and enteroendocrine-cell-specific *boss* knockdown flies reduced their stored lipids and food intake.

At present, few reports have shown that the input of a gene or signaling pathway from some tissue is counteracted by the input of that from another tissue. Cyclic AMP responsive element binding protein (CREB) is an evolutionarily conserved transcription factor involved in many important physiological functions [23]. CREB activity either in neurons or in the fat body controls energy stores and feeding behavior, but the effects are opposite [24]. Another example of this counteraction scenario is found in circadian clock genes. Flies lacking circadian clock (*clock* knockdown or dominant negative form of *cycle* overexpression) in the fat body display increased food intake but decreased stored glycogen and TAG. On the other hand, flies lacking circadian clocks in neurons display decreased food intake but increased stored glycogen and TAG [25]. Thus, it is an especially interesting issue to investigate the correlation between BOSS and CREB or circadian clocks in the future.

### BOSS is expressed in nutrient-sensing tissues

There is growing evidence that physiological communication between tissues coordinate food intake and utilization of nutrient stores with energy requirements [16]. For example, the brain functions as a key integrator of energy homeostasis that occurs in other tissues, such as in the fat body and in muscle [16, 26]. How do these tissues communicate with each other?

One of the major systems involved in the communication among tissues is the endocrine signaling network [2]. Hormones and neuropeptides—many of which are conserved between *Drosophila* and mammals—are secreted and used for inter-organ and inter-tissue communication [16]. Certain tissues are known to secret endocrine signals and transmit information about food or body energy condition. In the gut, enteroendocrine cells that are chemosensory cells, for example, secrete peptides in response to the nutritional status of the body [27]. In mammals and *Drosophila*, taste receptors are expressed in enteroendocrine cells, implying that these taste receptors have chemosensory roles in the regulation of physiological functions such as food intake, nutrient absorption, and sugar homeostasis [17, 28].

The fat body functions not only as an energy store organ but also as an endocrine organ. The fat body secrets TAG under starving conditions and functions to maintain energy homeostasis [29]. Recently, Upd2 was identified as a leptin homologue secreted from the fat body [30]. In *Drosophila*, many peptides that regulate feeding and energy metabolism have been identified, and many endocrine signals (hormones and neuropeptides) are conserved between *Drosophila* and mammals [31].

In this study, we demonstrated that neuronally expressed BOSS affects food intake and energy metabolism. In addition, we showed that BOSS is expressed in neuronal and peripheral tissues that also express neuropeptides and hormones (Fig 4). Thus, it will be of great interest to investigate in the future how BOSS regulates endocrine signals and coordinate energy metabolism.

We also found that BOSS is expressed in both gustatory and olfactory organs (Fig 4A–4C). Feeding behavior is determined by taste and other sensory information, such as smell, touch, vision, and ambient temperature. One report describes how odor affects taste behavior through the maxillary palp [32]. Thus, in this context, further detailed analysis of neuronal BOSS’s function will reveal more precise physiological roles of neuronal BOSS. It will be interesting to examine whether and how BOSS expressed in the olfactory organs, the antenna, and the maxillary palp modulates feeding behavior.

BOSS is an evolutionarily conserved protein [33]. The expression of GPRC5B, a mammalian BOSS homologue, is similar to that of BOSS [6, 7, 34]. GPRC5B is broadly expressed in brain, gut, and adipose tissues. Recently, aberrant expression of GPRC5B was identified as an obesity risk factor [9]. The metabolism of *Drosophila* and mammals shows similarities with evolutionarily conserved signaling pathways and analogous tissues that regulate energy metabolism [35]. Thus, specific analysis of *Drosophila* BOSS will open the door for understanding the broader physiological functions of the highly conserved membrane protein BOSS/GPRC5B.

## Supporting Information

S1 Fig Expression of *Drosophila insulin-like peptides* (*dilps*) in *boss* mutant flies. Expression of *Drosophila* insulins (*dilp2*, *3*, *5*, and *6*) were quantified by qRT-PCR (n = 3, 10 flies per replicate). Data are represented as mean ±SEM (*P<0.05).
(TIFF)

S2 Fig BOSS-expressing cells in the gut. (A) Expression of *boss* in the gut was visualized by expression of 10xUAS-mCD8.GFP using *boss-GAL4* driver. To mark enteroendocrine cells, the antibody against prospero was used. Arrowheads show the prospero-positive GFP-expressing cells. Scale bars; 100 μm. (B) Total number of GFP (BOSS)- and prospero-positive cells in the larval and adult midgut. Cells were counted from immunofluoresence images (n = 3 each)
(TIFF)

S3 Fig BOSS expression in tissue-specific *boss* knockdown (KD) flies. To confirm that *boss* knockdowns were effective, expression of boss mRNA was quantified by qRT-PCR (n = 3, 10 flies per replicate). (A) Neuronal-specific boss KD. (B) Enteroendocrine cells-specific boss KD. (C) Fat body-specific boss KD. To generate tissue specific *boss* KD flies, adult flies carrying different Gal4 driver [elav-Gal4 for neuronal KD, prospero-Gla4 for enteroendocrine cells KD and FB-Gal4 for fat body KD] were crossed to boss-RNAi lines [boss-RNAi#1(v4365) or boss-RNAi#2 (v4366)]. mRNA were collected from head for neuronal KD, whole gut for enteroendocrine cells KD, and body for fat body KD, since isolation of these tissues alone from whole flies was technically difficult. Data are represented as mean ±SEM (*P<0.05).
(TIFF)

S4 Fig BOSS expression in tissue-specific *boss* knockdown flies. (A) Summary of BOSS-expressing organs/tissues in larva and adult. (B) Schematic model proposed to explain the roles of BOSS in regulating food intake and energy storage. BOSS-expressing tissues sense nutrition (glucose) in food or hemolymph, induce hormones and neuropeptides for coordinating inter-organ communication (arrows), and maintain energy homeostasis. BOSS expression in neurons and gut promotes food intake and energy storage, but BOSS expression in fat body suppresses food intake and energy storage.
(TIFF)

S1 Table Primer sequences for qRT-PCR. (TIFF)
