# Supplementary figures and images for: Differential Effects of Tissue-Specific Deletion of BOSS on Feeding Behaviors and Energy Metabolism
Source: PLoS One. 2015 Jul 20;10(7):e0133083. doi: 10.1371/journal.pone.0133083 (PMC4508045; doi:10.1371/journal.pone.0133083)

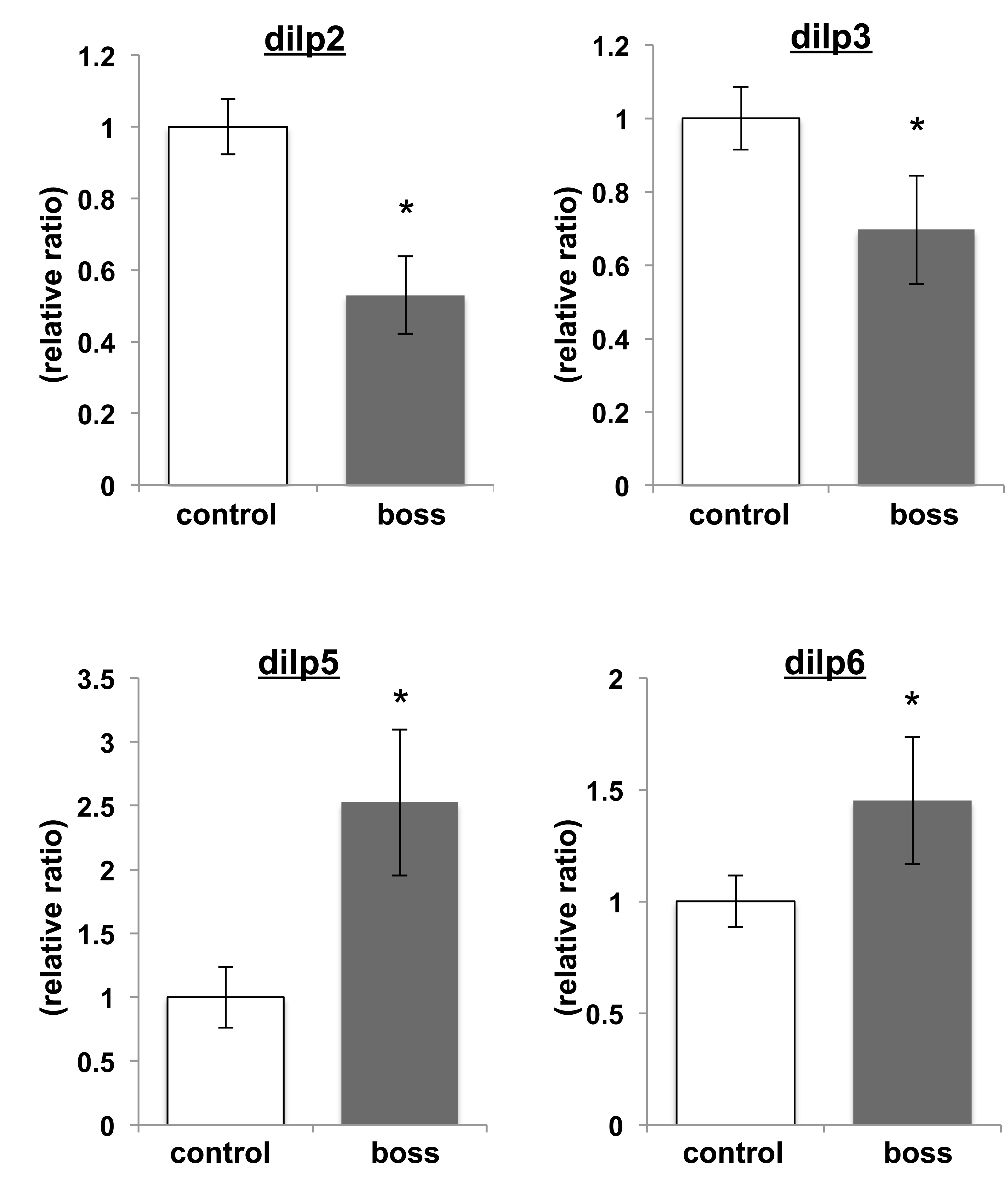

Supplement: S1 Fig — Expression of Drosophila insulins (dilp2, 3, 5, and 6) were quantified by qRT-PCR (n = 3, 10 flies per replicate). Data are represented as mean ±SEM (*P<0.05). (TIFF) [file pone.0133083.s001.tiff]

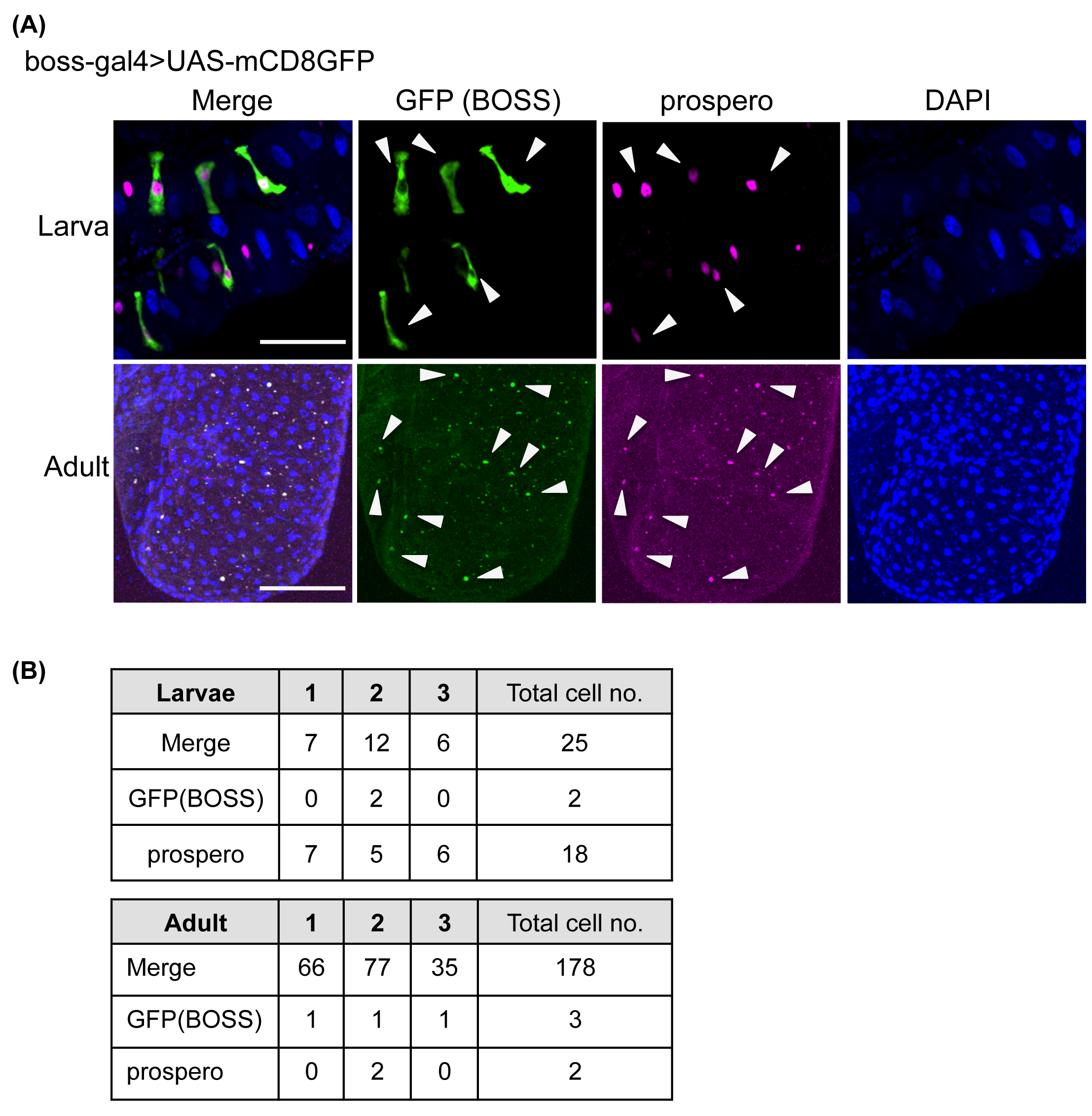

Supplement: S2 Fig — (A) Expression of boss in the gut was visualized by expression of 10xUAS-mCD8.GFP using boss-GAL4 driver. To mark enteroendocrine cells, the antibody against prospero was used. Arrowheads show the prospero-positive GFP-expressing cells. Scale bars; 100 μm. (B) Total number of GFP (BOSS)- and prospero-positive cells in the larval and adult midgut. Cells were counted from immunofluoresence images (n = 3 each) (TIFF) [file pone.0133083.s002.tiff]

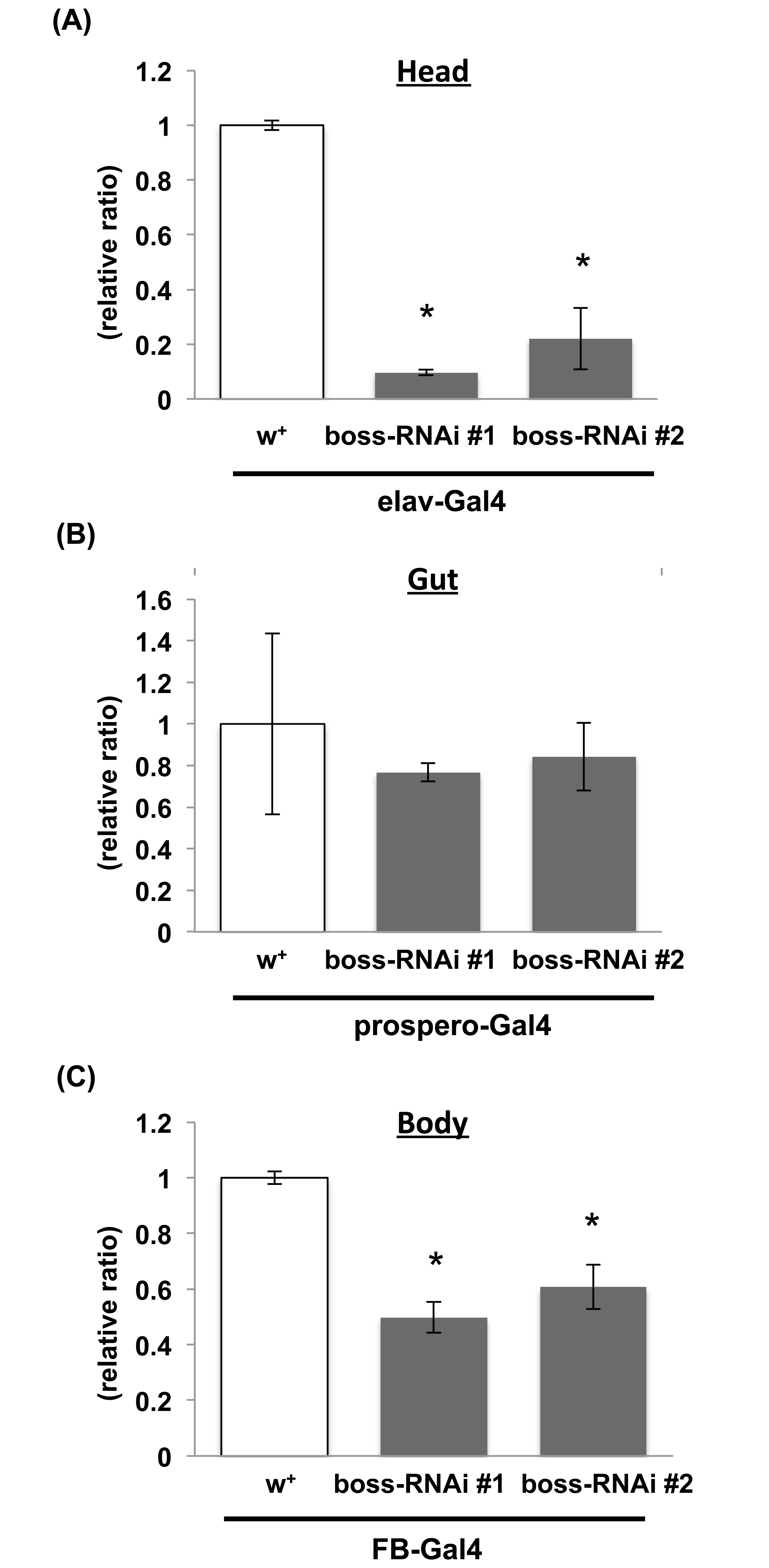

Supplement: S3 Fig — To confirm that boss knockdowns were effective, expression of boss mRNA was quantified by qRT-PCR (n = 3, 10 flies per replicate). (A) Neuronal-specific boss KD. (B) Enteroendocrine cells-specific boss KD. (C) Fat body-specific boss KD. To generate tissue specific boss KD flies, adult flies carrying different Gal4 driver [elav-Gal4 for neuronal KD, prospero-Gla4 for enteroendocrine cells KD and FB-Gal4 for fat body KD] were crossed to boss-RNAi lines [boss-RNAi#1(v4365) or boss-RNAi#2 (v4366)]. mRNA were collected from head for neuronal KD, whole gut for enteroendocrine cells KD, and body for fat body KD, since isolation of these tissues alone from whole flies was technically difficult. Data are represented as mean ±SEM (*P<0.05). (TIFF) [file pone.0133083.s003.tiff]

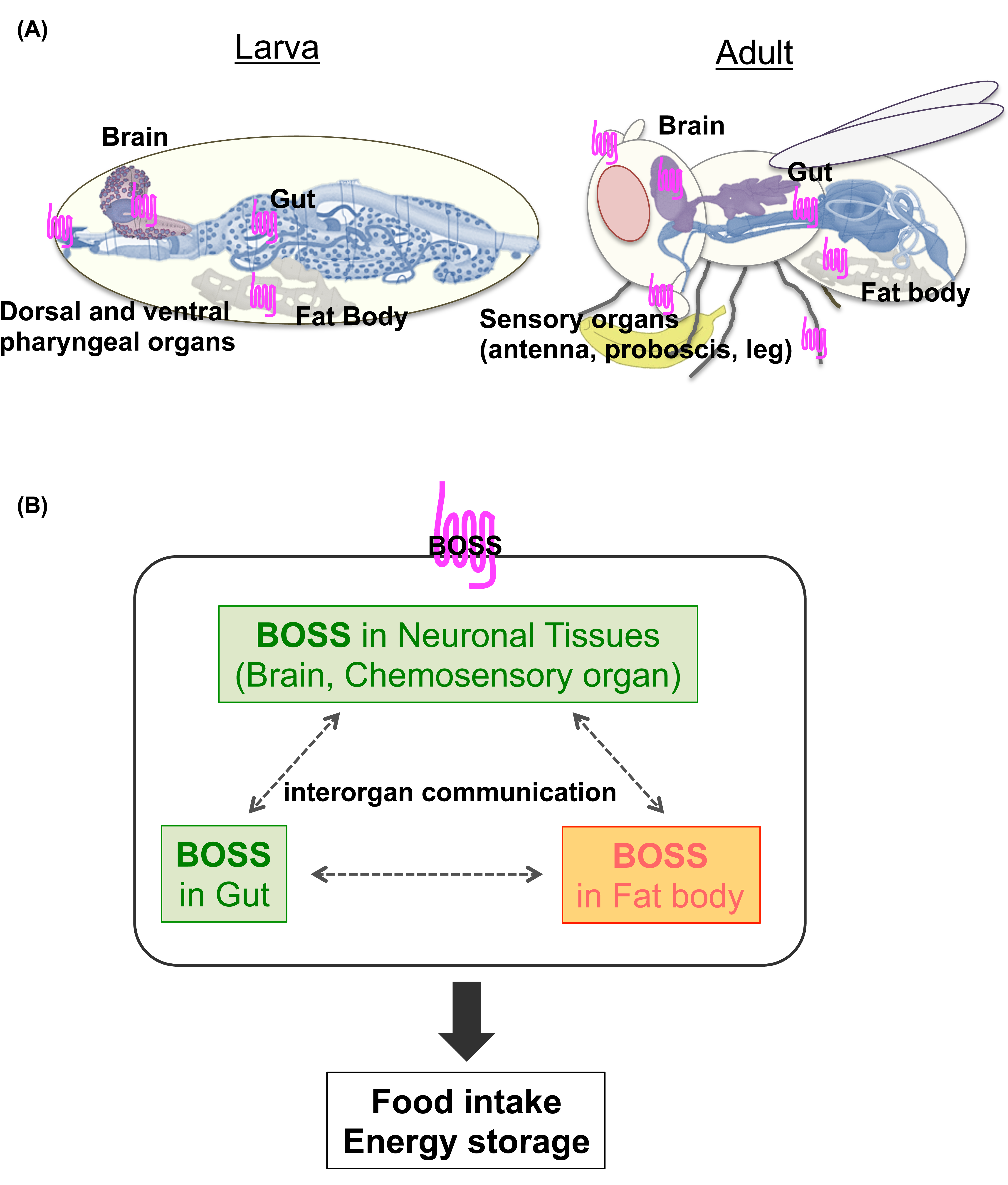

Supplement: S4 Fig — (A) Summary of BOSS-expressing organs/tissues in larva and adult. (B) Schematic model proposed to explain the roles of BOSS in regulating food intake and energy storage. BOSS-expressing tissues sense nutrition (glucose) in food or hemolymph, induce hormones and neuropeptides for coordinating inter-organ communication (arrows), and maintain energy homeostasis. BOSS expression in neurons and gut promotes food intake and energy storage, but BOSS expression in fat body suppresses food intake and energy storage. (TIFF) [file pone.0133083.s004.tiff]

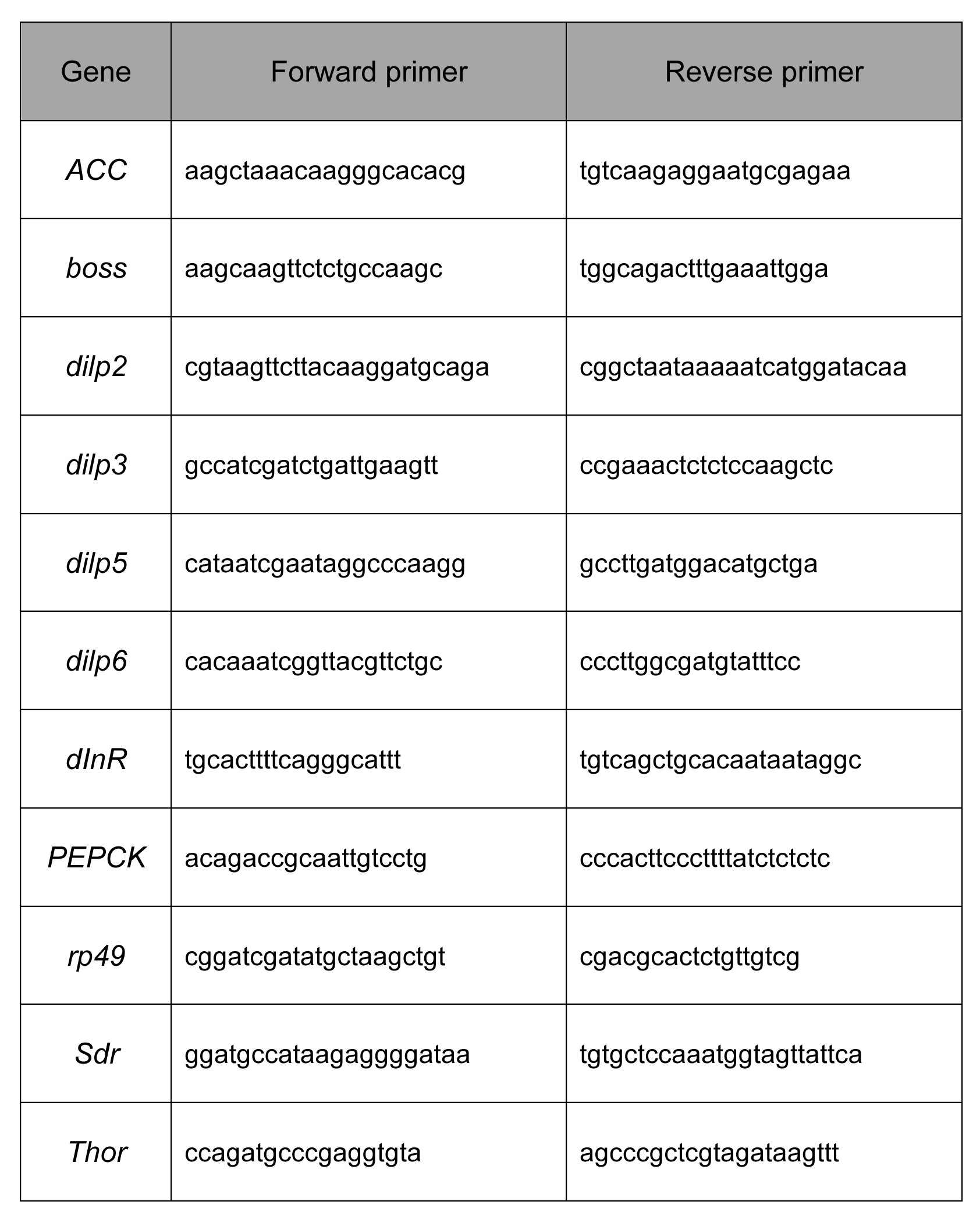

Supplement: S1 Table — (TIFF) [file pone.0133083.s005.tiff]
